# Design and Optimization of a Process for Sugarcane Molasses Fermentation by *Saccharomyces cerevisiae* Using Response Surface Methodology

**DOI:** 10.1155/2013/815631

**Published:** 2013-10-10

**Authors:** Nour Sh. El-Gendy, Hekmat R. Madian, Salem S. Abu Amr

**Affiliations:** ^1^Egyptian Petroleum Research Institute, Nasr City, Cairo 11727, Egypt; ^2^School of Civil Engineering, Engineering Campus, Universiti Sains Malaysia, 14300 Nibong Tebal, Penang, Malaysia

## Abstract

A statistical model was developed in this study to describe bioethanol production through a batch fermentation process of sugarcane molasses by locally isolated *Saccharomyces cerevisiae* Y-39. Response surface methodology RSM based on central composite face centered design CCFD was employed to statistically evaluate and optimize the conditions for maximum bioethanol production and study the significance and interaction of incubation period, initial pH, incubation temperature, and molasses concentration on bioethanol yield. With the use of the developed quadratic model equation, a maximum ethanol production of 255 g/L was obtained in a batch fermentation process at optimum operating conditions of approximately 71 h, pH 5.6, 38°C, molasses concentration 18% wt.%, and 100 rpm.

## 1. Introduction

There is an increased interest in alternative fuels, especially liquid transportation fuels. Bioethanol is one of the most employed liquid biofuels due to the easy adaptability of this fuel to existing engines and because this is a cleaner fuel with higher octane rating than gasoline [1]. Ethanol market grew from less than a billion liters in 1975 to more than 39 billion liters in 2006 and is expected to reach 100 billion liters in 2015 [2]. 

Among the widely used substrates for ethanol production are the molasses, the wastes byproduct of sugar industries from sugarcane and sugar beet. This is because they are cheap raw materials, readily available, and ready for conversion with limited pretreatments as compared with starchy or cellulosic materials, as all sugars are present in a readily fermentable form [3]. 

Yeasts are the most commonly used microorganisms for ethanol fermentation. *Saccharomyces cerevisiae* is one of the well-known ethanol producers [4].

Ongoing research and development seeking to improve methods by minimizing the numbers of experiments provide information about the direct additive effects of the study variables and interaction effects using design of experiment methods. Recently, this statistical technique has been successfully applied in many fields [5–8]. Response surface Methodology (RSM) is a combination of mathematical and statistical techniques and is used for the modeling and analysis of problems in which a response of interest is influenced by several variables, and the objective is to optimize this response [9]. The most popular RSM design is the central composite design (CCD) for analysis of experimental data. The CCD is applied to estimate the coefficients of a particular model equation. The CCD is efficient and flexible, providing sufficient information about the effects of variables and overall experimental error with a minimum number of experiments. Center points in CCD design are usually repeated 4–6 times to get a good estimate of experimental error (pure error). Five center points are created by default for each factor: alpha, with negative, zero and positive values (−1, 0, and 1) [9].

In this study, alpha value was taken as one resulting in 3 levels, lowest (−1), middle (0), and highest (+1) which is more specifically known as central composite face centered design (CCFD), in an attempt to optimize the variables: incubation period, initial pH, incubation temperature, and molasses concentration, which affect bioethanol production.

The main goal of the present work was to maximize bioethanol production from sugarcane molasses in batch fermentation process using previously isolated *Saccharomyces cerevisiae* Y-39. The application of CCFD and RSM assisted in designing, modeling, and optimizing the fermentation process by performing a series of controlled laboratory experiments.

## 2. Materials and Methods

### 2.1. Feedstock

Sugarcane molasses was purchased from Sugars and Integrated Industries Egyptian Distillation Plants in Hawamdeia City, Giza, Egypt and stored at −4°C until use.

### 2.2. Media

Wickerham WH medium prepared according to Wickerham [10] was used for maintenance and inoculum preparation.

Medium for fermentation experiments was prepared as follows: 2 g KH_2_PO_4_, 10 g (NH_4_)_2_SO_4_, 1 g MgSO_4_ · 7H_2_O, and 2 g yeast extract were dissolved in 1 L distilled water, molasses concentration and pH were then adjusted according to the experimental conditions before sterilization, at 121°C for 20 min to avoid contamination.

### 2.3. Microorganism and Inoculum Preparation

A yeast strain *Saccharomyces cerevisiae* Y-39, previously isolated for its ability to produce bioethanol from different saccharides [11], was used in this study. Active cultures for fermentation experiments were prepared by growing Y-39 in WH medium for 48 h at 30°C in shaking incubator 150 rpm. Harvested cells were washed twice with sterile saline (8.5 g NaCl per 1 L distilled water) and then resuspended in sterile saline to be used as a fresh and pure stock for inoculation.

### 2.4. Analytical Methods

The types of sugars in molasses were determined by high performance liquid chromatography (HPLC), according to the method reported by Madian et al. [12]. Ashes were quantified by gravimetric analysis after burning samples at 550°C for 3 h, and minerals concentrations were determined by atomic absorption spectrophotometer in Central Analytical Lab. in Egyptian Petroleum Research Institute. All other chemical characterizations of molasses were done in Agricultural Research Center, Giza, Egypt. Ethanol yield was measured by Gas chromatography (model 6890, Agilent), equipped with flame ionization detector and nominal capillary column (60 m × 530 *μ*m × 5.00 *μ*m). Helium was the carrier gas; flow rate was 25 mL/min. Oven and detector temperatures were 300°C. All experiments were carried out in triplicates, and the listed results are the average.

### 2.5. Fermentation Experiments

Batch fermentations were done in 100 mL Erlenmeyer flasks fitted with rubber stoppers, containing 50 mL of culture media, inoculated with 5 mL of fresh yeast inoculum stock (*≈*10 mg fresh yeast/mL). Incubation was performed in shaking incubator 100 rpm, set at temperatures according to the required experimental conditions. Samples for analyses were taken at the beginning and end of fermentation at the prescribed incubation periods. 

### 2.6. Experimental Design

Response surface methodology (RSM) was used to optimize bioethanol production process from sugarcane molasses and investigate the influence of different fermentation process variables on the bioethanol yield. The central composite face centered design CCFD was applied to study process variables. The experimental runs were carried out according to a 2^4^ full factorial design for the four identified design independent variables, namely, incubation period h (*X*_1_), initial pH (*X*_2_), incubation temperature, °C (*X*_3_), and molasses concentration wt.% (*X*_4_), with low (−1) and high (+1) levels. The total number of experiments (runs) was given by the simple formula [30 = 2^*k*^ + 2*k* + 6], where *k* is the number of independent variables (*k* = 4), this includes 16 factorial points from 24 full factorial CCFD were augmented with 6 replicates at the center point to assess the pure error. Response selected was bioethanol yield. The levels were selected based on preliminary study results. The design factors (variables) with low −1 and high +1 levels, are, namely, *X*_1_ [24 and 72 h], *X*_2_ [5 and 7], *X*_3_ [20 and 40°C], and *X*_4_ [15 and 25 wt.%]. The central values; zero level chosen for experimental design were 48 h, 6, 30°C, and 20% for *X*_1_, *X*_2_, *X*_3_, and *X*_4_, respectively (Table 1).

### 2.7. Statistical Analysis

Once the experiments were preformed, the next step was to perform a response surface experiment to produce a prediction model to determine curvature, detect interactions among the design factors (independent variables), and optimize the process, that is, determine the local optimum independent variables with maximum yield of bioethanol. The model used in this study to estimate the response surface is the quadratic polynomial represented by the following equation:

(1) $$\begin{array}{c} Y=\beta_{o}+\sum_{i=1}^{4}\beta_{i}x_{i}+\sum_{i=1}^{3}\sum_{j=i+1}^{4}\beta_{ij}x_{i}x_{j}+\sum_{i=1}^{4}\beta_{ii}x_{i}^{2}, \end{array}$$

where *Y* is the bioethanol yield (g/L), *β*_*o*_ is the value of the fixed response at the center point of the the design, *β*_*i*_, *β*_*ij*_, and *β*_*ii*_ are the linear, interactive, and quadratic coefficients, respectively. *x*_*i*_ and *x*_*j*_ are the independent variables (factors) under study. 

The statistical software Design Expert 6.0.7 (Stat-Ease Inc., Minneapolis, USA) was used for design of experiments, regression and graphical analyses of the data obtained, and statistical analysis of the model to evaluate the analysis of variance (ANOVA).

## 3. Results and Discussion

### 3.1. Chemical Composition of Molasses

Sugarcane molasses is a dark viscous fluid with pH value of 5 and very rich in nutrients required by most microorganisms. Carbon, nitrogen, phosphorus, sodium, and potassium contents are 64, 6, 0.3, 0.33, and 5.5 (wt.%), respectively. Non-nitrogenous compounds (e.g., citric acid, oxalic acid) represent 2–8% (wt.%). Molasses has no furfural which is toxic to most of fermenting microorganisms. The ashes (11% wt.%) constitute a source of mineral elements. Molasses is found to be rich in calcium *≈*0.7% and contains significant quantities of trace minerals copper (2.2 ppm), zinc (3.91 ppm), manganese (4.74 ppm), iron (78.37 ppm), and magnesium (1370 ppm). It is composed of 68.36% sucrose, 18.50% glucose, and 13.14% maltose. Sugarcane molasses is rich in fermentable sugars *≈*55% (wt%) and non-fermentable sugars recorded *≈*5% (wt%).

Most of the chemical parameters determined in this study were in close agreement to those reported by Chen and Chou [13], who found molasses containing 45–55% total sugars, 20–25% reducing sugars, 25–35% sucrose, 10–16% ash, 0.4–0.8% calcium, 0.1–0.4% sodium, 1.5–5% potassium and pH 5–5.5.

Soil and climate, the variety and maturity of the cane, and the processing conditions in the factory all influence molasses composition. Consequently, considerable variation may be found in nutrient content, flavor, color, and viscosity of molasses. But generally, sucrose is the major sugar present.

### 3.2. Optimization of Transesterification Process and Interaction between Independent Variables

Based on CCFD and experimental results, RSM was used to optimize fermentation process design factors (independent variables). The statistical combinations of variables in coded and actual values along with the predicted and experimental responses are presented in Table 2. The regression equation characterizing the influence of different considered variables on process yield was obtained:

(2) Y=177.4+6.34X1−0.41X2−3.33X3−2.39X4 −33.32X1X2+28.51X1X3−22.11X1X4 −27.60X2X3+30.38X2X4−16.39X3X4 −0.56X12−92.58X22+44.08X32−66.47X42.

**Figure 1:**
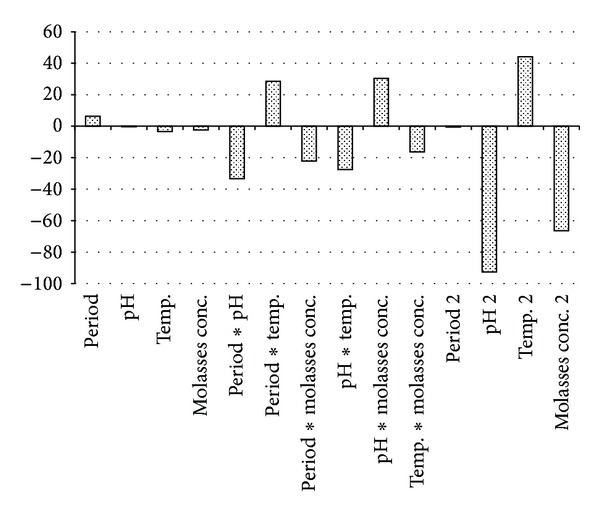
Pareto chart showing the effects of different independent variables on bioethanol yield.

Positive sign in front of the terms indicate synergetic effect, whereas negative sign indicates antagonistic effect. Pareto chart Figure 1, was used in this work to make it much easier to visualize the main and interaction effects of all factors to the response variable, that is, bioethanol yield. The model identified that within the studied range of experiments, the quadratic effect of incubation temperature and the interactive effect of initial pH and molasses concentration and that of incubation period and temperature have highly significant positive influence on the bioethanol yield. That is, with increment of both incubation period and temperature, the bioethanol yield increases, and the same occurred with increment of both pH and molasses concentration. But incubation period has a relatively low significant positive effect on bioethanol yield, while the quadratic effect of initial pH and molasses concentration, and the interactive effect of incubation period and initial pH as well as interactive effect of incubation temperature and initial pH have highly significant inverse effect on the bioethanol yield. The interactive effect of incubation period and molasses concentration and that of incubation temperature and molasses concentration have a significant negative impact on the bioethanol yield. Initial pH, incubation temperature, molasses concentration, and the quadratic effect of incubation period seem to have negative impact on bioethanol yield. Thus, bioethanol yield decreases with increase of the initial pH and incubation period, incubation temperature and initial pH, incubation period and molasses concentration or incubation temperature and molasses concentration. 

#### 3.2.1. Statistical Analysis and Validation of Model

The validity of the fitted model was evaluated, and its statistical significance was controlled by *F*-test. The analysis of variance (ANOVA) for the response surface full quadratic model is given in Table 3. It can be indicated that the model is highly statistically significant at 95% confidence level, with *F*-value of 82.14, and very low probability *P* value of < 0.0001. The values of the determination coefficients, *R* ^2^  and  *R*_adj_^2^ which measure the model fitting reliability for model (2), were calculated to be 0.9871 and 0.9751, respectively. This suggests that approximately 98.71% of the variance is attributed to the variables and indicated a high significance of the model. Thus, only 0.0129 of the total variations cannot be explained by the model which ensures the good adjustment of the above model to experimental data. Confirmation of the adequacy of the regression model was reflected also by the good agreement between experimental and predicted values of response variables as shown in Table 2. Where, the actual bioethanol yield ranged from 5 to 243.32 g/L and its corresponding predicted values are 1.98 and 224.85 g/L, respectively. “Adeq Precision” measures the signal to noise ratio. A ratio greater than 4 is desirable. The ratio of 27.282 indicated an adequate signal. This model is reliable and can be used to navigate the design space. The lack of fit test is performed by comparing the viability of the current model residuals to the variability between observations at replicate settings of the factors. The lack of fit was statistically significant with *F*-value of 53.24 and *P* value of 0.0002. A significant lack of fit suggests that there may be some systematic variation unaccounted for in the hypothesized model. This may be due to the exact replicate values of the independent variable in the model that provide an estimate of pure error. 

**Figure 2:**
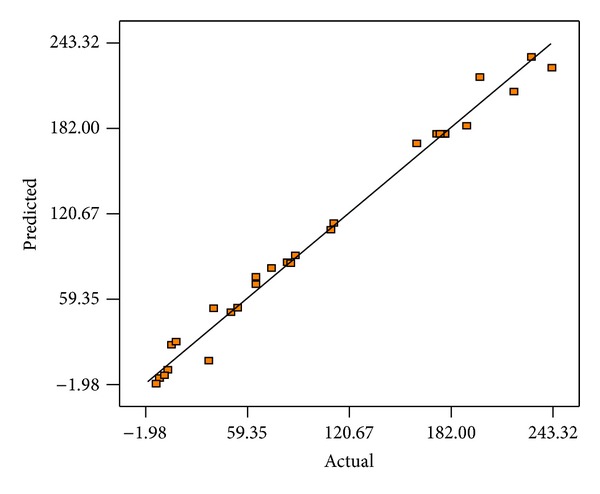
Experimental values versus predicted values for the model.

The relationship between predicted and experimental values of bioethanol yield is shown in Figure 2. It can be seen that there is a high correlation (*R* ^2^ = 0.9853) between the predicted and experimental values indicating that the predicted and experimental values were in reasonable agreement. It means that the data fit well with the model and give a convincingly good estimate of response for the system in the experimental range studied. 

**Figure 3:**
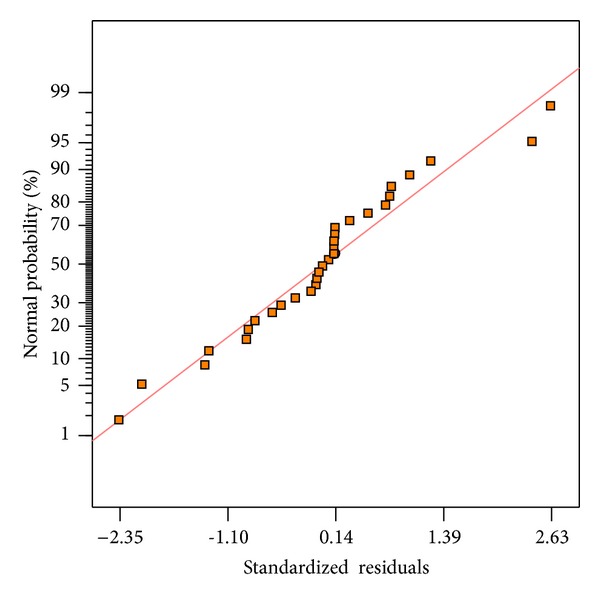
Normal probability plot of the residuals.

Figure 3 shows the normal probability plots of the standardized residuals for bioethanol production efficiency. A normal probability plot indicates that if the residuals follow a normal distribution, in which case the points will follow a straight line. Since some scattering is expected even with the normal data, as shown in Figure 3, it can be assumed that the data is normally distributed. Thus, the obtained normal probability plot indicates a good validity for the approximation of the quadratic regression model. 

**Figure 4:**
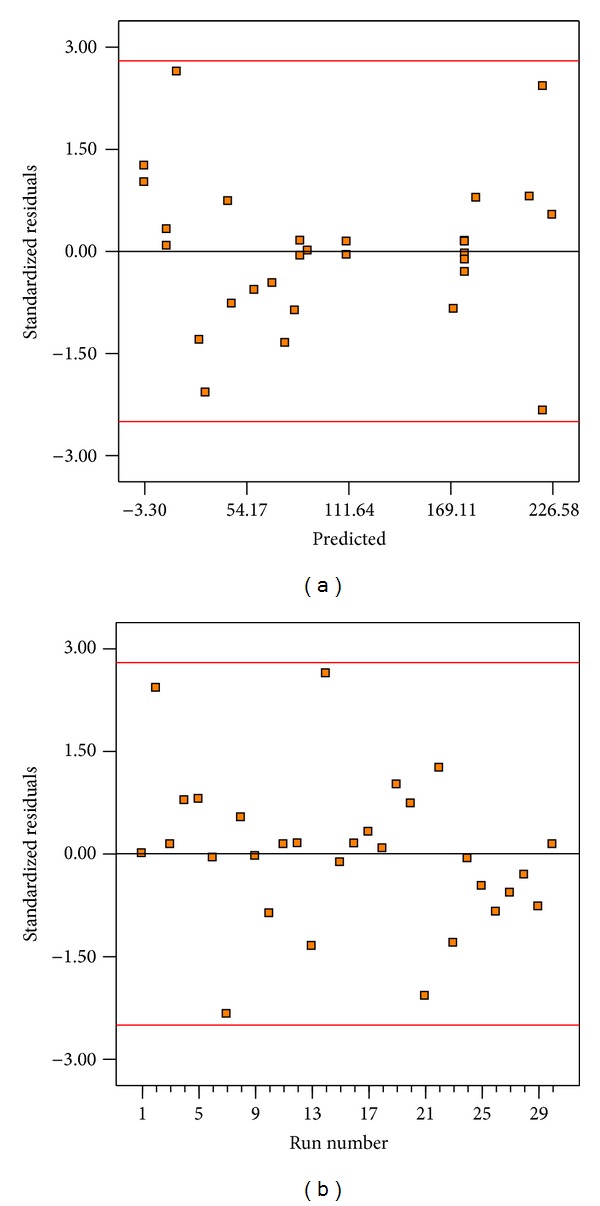
Diagnostic plots for bioethanol yield (a) residual versus predicted yield and (b) residual versus run number.

Figure 4(a) shows residual versus predicted values for bioethanol yield. In this research, points of observed runs were scattered randomly within the constant range of residuals across the graph. Thus, it revealed no obvious pattern and unusual structure. That is, the model is adequate, and there is no reason to suspect any violation of the independence or constant variance assumption in all runs. The standardized residuals versus run plot represented in Figure 4(b) shows randomly scattered points ranged between ±2.8; the errors were normally distributed and insignificant. 

**Figure 5:**
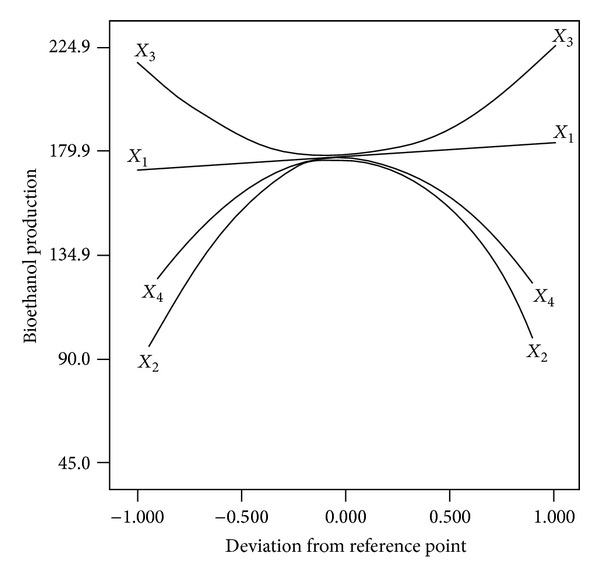
Perturbation plot for bioethanol yield.

The perturbation plot Figure 5 shows the comparative effects of all independent variables on bioethanol yield. The sharp curvature of the three factors: initial pH, incubation temperature, and molasses concentration shows that the response, bioethanol yield, was very sensitive to these three variables. The comparatively semiflat incubation period curve shows less sensitivity of bioethanol yield towards the incubation period. Thus, the incubation period with the studied range of experiments has no major function in the fermentation process compared to the other three factors.

#### 3.2.2. Interaction among Factors Influencing Fermentation Process and Bioethanol Yield

**Figure 6:**
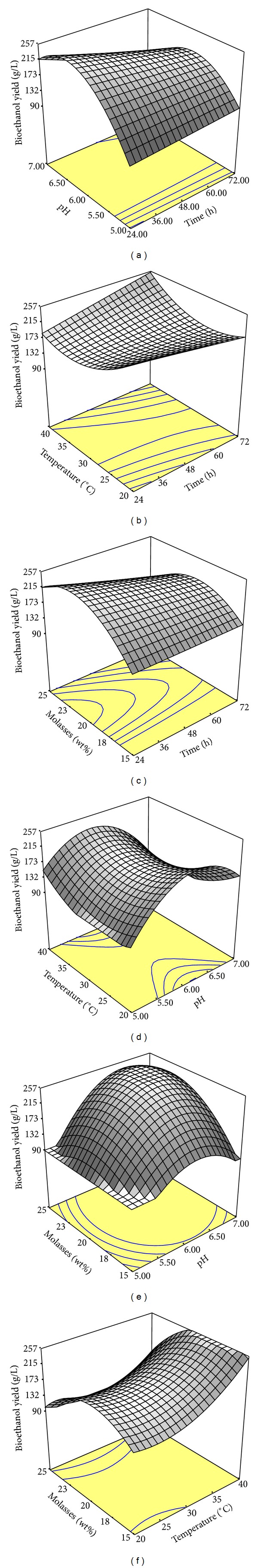
Response surface plots of bioethanol yield.

The empirical predicted quadratic model for response (bioethanol yield) in terms of process variables (incubation period, initial pH, incubation temperature, and molasses concentration) are plotted in three-dimensional diagrams (Figure 6), to investigate the interaction among the variables and to determine the optimum condition of each factor for maximum bioethanol yield.

Figure 6(a) represents the effects of varying, incubation period and initial pH on bioethanol yield at constant incubation temperature 38°C and molasses concentration 18 wt%. It is obvious that the initial pH has more powerful effect than incubation period. However, increase in bioethanol yield occurred with an increase in incubation period at pH range from 5.5 to 6.5. Further increase in initial pH would decrease the bioethanol yield. According to this interaction effects, the maximum yield of bioethanol was *≈*257 g/L at *≈*pH 6 and 72 h incubation period. 

Figure 6(b) shows the effect of both incubation period and temperature on bioethanol yield g/L at constant initial pH 5.6 and molasses concentration 18 wt%. A significant positive impact is detectable; that is, bioethanol yield increased with the increase of incubation temperature and time. Nevertheless, incubation temperature exhibits a more powerful effect than that of incubation period. It is obvious that the maximum yield of bioethanol was *≈*257 g/L at higher incubation period *≈*72 h and temperature *≈*40°C. 

Figure 6(c) shows the interactive effect of incubation period h and molasses concentration wt.% on bioethanol production at constant initial pH 5.6 and incubation temperature 38°C. It is obvious that with the increase of molasses concentration above *≈*20 wt%, bioethanol yield decreases. According to this figure, the maximum bioethanol production *≈*254 g/L obtained at higher incubation period *≈*72 h and 20 wt.% molasses concentration. 

Figure 6(d) shows the cooperative effect of incubation temperature and initial pH on bioethanol yield at constant incubation period 72 h and molasses concentration 18 wt%. As shown in Figure 6(d), at low and high pH values, the bioethanol yield decreases. The maximum bioethanol yield *≈*257 g/L was obtained at high incubation temperature *≈*40°C and initial pH of *≈*6. 

Figure 6(e) illustrates the interactive effect of initial pH and molasses concentration on bioethanol yield at constant incubation period 72 h and temperature 40°C. The elliptical shape of the curve indicates a strong interaction between the variables. The positive interactive effect of initial pH and molasses concentration is obvious. Where, bioethanol yield is low at both low and high molasses concentrations and initial pH. But bioethanol yield increases within pH range 5.5–6.5 and molasses concentration 18%–22% with maximum yield of *≈*257 g/L at pH and molasses concentration of *≈*6 and 20%, respectively. 

Figure 6(f) represents the interactive effect of incubation temperature and molasses concentration on bioethanol yield at constant incubation period 72 h and initial pH 5.6. It is obvious that with the increase in temperature, the bioethanol yield increases reaching its maximum at *≈*40°C, while at low and high molasses concentrations the yield decreases and increased within 18%–22%. The maximum bioethanol yield of *≈*257 g/L is obtained at molasses concentration and incubation temperature of *≈*20% and 40°C. 

#### 3.2.3. Optimization of Fermentation Process and Model Verification

The optimization process was carried out to determine the optimum value of bioethanol production efficiency, using the Design Expert 6.0.7 software. According to the software optimization step, the desired goal for each operational condition (*X*_1_ incubation period, *X*_2_ initial pH, *X*_3_ incubation temperature, and *X*_4_ molasses concentration) was chosen “within” the studied range. The response (bioethanol production) was defined as maximum to achieve the highest performance. The program combines the individual desirability into a single number and then searches to optimize this function based on the response goal. Accordingly, the optimum working conditions and respective bioethanol production were established, and the results are presented in Table 4. An additional experiment was then performed to confirm the optimum results. As shown, the maximum bioethanol production was *≈*255 g/L at incubation period of *≈*71 h, initial pH *≈*5.6, incubation temperature *≈*38°C, and molasses concentration of *≈*18%. The desirability function value was found to be 1.000 for these optimum conditions. The laboratory experiment agrees well with the predicted response value *≈*253 g/L.

Standard deviation and percent error were calculated for validation of experiments. Recording average of *≈*1.08 and 0.87%, respectively, indicating that process optimization by CCFD was capable and reliable to optimize bioethanol production from sugarcane molasses using *Saccharomyces cerevisiae* Y-39. 

## 4. Conclusion

RSM and CCFD proved to be reliable and powerful tool for modeling, optimizing and studying the interactive effects of four process variables (incubation period, initial pH, incubation temperature, and molasses concentration) of bioethanol production from batch fermentation of sugarcane molasses using locally isolated* Saccharomyces cerevisiae* Y-39. A highly significant (*R* ^2^ = 0.9871, *P* < 0.0001) regression quadratic model equation was obtained by analyzing the data of a 2^4^ factorial design. The maximum predicted and actual bioethanol yields are 253 and 255 g/L, respectively.

## Figures and Tables

**Table 1:** Variables and their levels used in the experimental design.

Variables	Symbol coded	Range and levels		
		Low level (−1)	Center (0)	High level +1
Incubation period h	*X*_1_	24	48	72
pH	*X*_2_	5	6	7
Incubation temperature °C	*X*_3_	20	30	40
Molasses concentration wt.%	*X*_4_	15	20	25

**Table 2:** Experimental design matrix with experimental and predicted values of bioethanol yield.

Run number	Factors/Levels									
	Incubation period h		Initial pH		Incubation temperature °C		Molasses conc wt.%		Bioethanol yield	
	*X*_1_		*X*_2_		*X*_3_		*X*_4_		g/L	
	Coded value	Actual value	Coded value	Actual value	Coded value	Actual value	Coded value	Actual value	Experimental data	Predicted data
1	+1	72.00	−1	5.00	+1	40.00	+1	25.00	88.86	90.14
2	0	48.00	0	6.00	+1	40.00	0	20.00	243.32	224.85
3	0	48.00	0	6.00	0	30.00	0	20.00	178.89	177.44
4	+1	72.00	0	6.00	0	30.00	0	20.00	192.03	183.22
5	−1	24.00	+1	7.00	−1	20.00	+1	25.00	220.45	207.73
6	0	48.00	0	6.00	0	30.00	+1	25.00	110.22	108.58
7	0	48.00	0	6.00	−1	20.00	0	20.00	200.00	218.18
8	+1	72.00	−1	5.00	+1	40.00	−1	15.00	230.93	232.68
9	0	48.00	0	6.00	0	30.00	0	20.00	177.00	177.44
10	+1	72.00	−1	5.00	−1	20.00	−1	15.00	74.39	81.02
11	0	48.00	0	6.00	0	30.00	−1	15.00	112.00	113.36
12	0	48.00	+1	7.00	0	30.00	0	20.00	86.00	84.45
13	−1	24.00	+1	7.00	−1	20.00	−1	15.00	65.00	74.75
14	−1	24.00	−1	5.00	−1	20.00	−1	15.00	36.73	14.50
15	0	48.00	0	6.00	0	30.00	0	20.00	176.00	177.44
16	0	48.00	0	6.00	0	30.00	0	20.00	179.00	177.44
17	+1	72.00	+1	7.00	−1	20.00	−1	15.00	12.00	8.01
18	+1	72.00	−1	5.00	−1	20.00	+1	25.00	10.00	4.04
19	−1	24.00	−1	5.00	+1	40.00	+1	25.00	5.00	1.98
20	+1	72.00	+1	7.00	+1	40.00	−1	15.00	50.00	49.27
21	−1	24.00	−1	5.00	−1	20.00	+1	25.00	14.18	25.95
22	−1	24.00	+1	7.00	+1	40.00	−1	15.00	7.00	1.99
23	+1	72.00	+1	7.00	+1	40.00	+1	25.00	17.00	28.27
24	0	48.00	−1	5.00	0	30.00	0	20.00	84.00	85.26
25	−1	24.00	+1	7.00	+1	40.00	+1	25.00	65.00	69.41
26	−1	24.00	0	6.00	0	30.00	0	20.00	162.00	170.53
27	+1	72.00	+1	7.00	−1	20.00	+1	25.00	54.00	52.56
28	0	48.00	0	6.00	0	30.00	0	20.00	174.00	177.44
29	−1	24.00	−1	5.00	+1	40.00	−1	15.00	39.66	52.13
30	0	48.00	0	6.00	0	30.00	0	20.00	178.90	177.44

**Table 3:** Analysis of variance of the fitted quadratic regression model (2).

Source	SS*	df*	MS*	*F* value	*P* value	Remarks
Model	1.701*e* + 005	14	12152.97	82.14	<0.0001	Significant
X_1_	724.41	1	724.41	4.90	0.0428	Significant
X_2_	2.96	1	2.96	0.020	0.8894	Not significant
X_3_	200.13	1	200.13	1.35	0.2630	Not significant
X_4_	102.72	1	102.72	0.69	0.4178	Not significant
X_1_X_2_	17759.56	1	17759.56	120.03	<0.0001	Significant
X_1_X_3_	13001.70	1	13001.70	87.87	<0.0001	Significant
X_1_X_4_	7818.98	1	7818.98	52.85	<0.0001	Significant
X_2_X_3_	12188.16	1	12188.16	82.38	<0.0001	Significant
X_2_X_4_	14769.54	1	14769.54	99.82	<0.0001	Significant
X_3_X_4_	4298.11	1	4298.11	29.05	<0.0001	Significant
X_1_^2^	0.83	1	0.83	5.585*e* − 003	0.9414	Not significant
X_2_^2^	22206.70	1	22206.70	150.09	<0.0001	Significant
X_3_^2^	5034.32	1	5034.32	34.03	<0.0001	Significant
X_4_^2^	11447.22	1	11447.22	77.37	<0.0001	Significant
Residual	2219.36	15	147.96			
Lack of Fit	2198.71	10	219.87	53.24	0.0002	Significant
Pure Error	20.65	5	4.13			

*SS: sum of squares; df: degree of freedom; MS: mean square.

**Table 4:** Optimum conditions solutions for bioethanol production.

Number of trials	Incubation period h	Initial pH	Incubation temperature °C	Molasses conc wt%	Desirability	Bioethanol yield g/L		Percent error	StD*
						Predicted	experimental		
1	67.54	5.88	39.21	20.43	1.000	245.1	248	1.18	1.45
2	26.79	6.35	20.31	22.89	1.000	245.2	247	0.73	0.9
3	25.00	6.21	20.39	21.84	1.000	248.8	250	0.48	0.6
4	71.25	5.59	38.14	17.52	1.000	252.7	255	0.9	1.15
5	71.05	5.36	39.41	20.23	1.000	247.5	249	0.6	0.75
6	26.74	6.15	20.20	20.68	1.000	245.5	248	1.02	1.25
7	30.11	6.39	20.23	22.02	1.000	244.1	247	1.18	1.45

*StD: standard deviation.
